# Sex differences of neural connectivity in internet gaming disorder and its association with sleep quality: an exploratory fMRI study

**DOI:** 10.3389/fpsyt.2024.1379259

**Published:** 2024-05-30

**Authors:** Mingzhe Zhou, Guoqing Gao, Bei Rong, Haomian Zhao, Junhua Huang, Ning Tu, Lihong Bu, Ling Xiao, Gaohua Wang

**Affiliations:** ^1^Department of Psychiatry, Renmin Hospital of Wuhan University, Wuhan, Hubei, China; ^2^Institute of Neuropsychiatry, Renmin Hospital of Wuhan University, Wuhan, Hubei, China; ^3^PET-CT/MR Center, Renmin Hospital of Wuhan University, Wuhan, Hubei, China; ^4^Taikang Center for Life and Medical Sciences, Wuhan University, Wuhan, Hubei, China

**Keywords:** internet gaming disorder, sex, regional homogeneity, functional connectivity, sleep quality

## Abstract

**Objectives:**

Sex-specific differences in internet gaming disorder (IGD) neurophysiology remain underexplored. Here we investigated sex-related variability in regional homogeneity (ReHo) and functional connectivity (FC) in IGD and their correlations with sleep quality.

**Methods:**

Resting-state functional magnetic resonance imaging (fMRI) scans were performed on 52 subjects with IGD and 50 healthy controls (HCs). Two-way ANOVA was used to examine sex and diagnosis interactions in ReHo and FC, followed by post-hoc analyses to explore FC biomarkers for different sexes.

**Results:**

In ReHo analysis, the four groups showed significant sex and diagnosis interactions in the right middle frontal gyrus (rMFG). FC analysis with rMFG as the seed region revealed a significant sex and diagnosis interaction effect in FC of the rMFG with the bilateral postcentral gyrus (PoCG). In male IGD group, FC between the rMFG and the bilateral PoCG correlates strongly with daytime dysfunction score and the Pittsburgh sleep quality inventory (PSQI) total score.

**Conclusion:**

These findings emphasize the importance of considering sexual dimorphism in the neurobiology of IGD, which might influence subsequent treatment strategies.

## 1 Introduction

Online gaming is now enjoyed by many for leisure and entertainment, but when it becomes continuous or repetitive, it can impair social functioning and/or produce clinical symptoms, potentially evolving into internet gaming disorder (IGD) (1, 2). The existence of IGD is still debated as a standalone psychiatric condition (3, 4). Nevertheless, the American Psychiatric Association lists IGD in the fifth edition of the Diagnostic and Statistical Manual of Mental Disorders (DSM-5) as a “condition requiring additional research” (5, 6), and the World Health Organization (WHO) has officially classified gaming disorder as a disease (7). Notably, during the COVID-19 pandemic, there was a significant increase in internet and gaming activities among children and adolescents across almost all Asian regions (8). IGD is increasingly recognized as a pressing and global socio-psychological concern.

IGD has a complex neurobiological basis, and is currently explained by several hypotheses, including the dual systems theory and the biopsychosocial model. The dual systems theory suggests that IGD involves an overactivation of the reward system and a weakening of the inhibitory control system, leading to impulsive and uncontrollable gaming behavior patterns (9). The biopsychosocial model emphasizes the combined effects of biological factors (such as genetic predispositions and changes in brain structure and function), psychological factors (including emotional states, personality traits, and cognitive patterns), and sociological factors (such as family environment, social relationships, and cultural background) on individuals with IGD, contributing to the development and persistence of addictive behaviors (10). Functional magnetic resonance imaging (fMRI) is a pivotal tool for studying brain function and plays a key role in investigating the neural mechanisms underlying IGD. While task-state fMRI has commonly been used in IGD research, resting-state (rs)-fMRI is gaining traction due to its high data reproducibility, extensive coverage, and straightforward, safe methodology (11, 12). ReHo quantifies the synchrony of a given voxel’s time series with its immediate neighbors through a measure known as Kendall’s coefficient of concordance (KCC) (13), and it is predominantly used as a metric representing the brain’s local coherence. FC links spatial regions of interest via linear time correlation (14). In recent studies, Niu et al. (15) applied both static and dynamic ReHo to male IGD individuals and found compromised connectivity within the frontal-striatal-thalamic circuit. Moreover, a longitudinal study of ReHo markers during natural recovery from IGD highlighted the critical involvement of specific brain regions including the dorsolateral prefrontal cortex (DLPFC), orbitofrontal cortex (OFC), and superior frontal gyrus (SFG), which are linked to reward and inhibitory control processes (16). Current research on FC has thoroughly explored the impact of IGD on dopamine reward system processing (17, 18), decision-making cognition (19), self-control (20), and attentional bias (21). Brain regions implicated in the pathophysiology of IGD often reflect deficits in self-monitoring, attention, interoception, motor control, and auditory processing, with its development and maintenance involving complex networks such as the default mode network (DMN), executive control network, and salience network (22, 23).

IGD is associated with a range of symptoms, and it coexists with other psychological or psychiatric conditions, including sleep disorders, anxiety, and depression (24–27). Excessive engagement in online gaming frequently precipitates adverse health outcomes, notably sleep-related issues (28). It is both a consequence and a contributing factor to mental disorders associated with excessive gaming. A previous exploratory study found individuals with internet addiction suffer disproportionately from poor sleep, constituting about 60% of the surveyed demographic (29). Furthermore, post-social media usage disorders, such as social media fatigue and social media addiction, have been implicated in deteriorating sleep quality (30). Additionally, the level of sleep quality also affects the severity of an individual’s IGD. University students with suboptimal sleep quality tend to exhibit more severe IGD, particularly amongst adolescents (31, 32). Poor sleep quality can exacerbate both poor health and social dysfunction, including diminished academic engagement (33). There are few robust studies on correlations between altered brain FC and sleep quality in different sexes within the IGD population, underscoring the need for a comprehensive analysis of these complex interactions to further our understanding of the multifaceted impact of IGD on brain pathophysiology.

IGD is associated with sex-specific variations in a spectrum of psychological traits — impulsivity, lack of self-control, anxiety, emotional instability, and depression — all of which are intricately linked to sleep quality (34). Further exploration into the manifestation of IGD across different sexes becomes particularly important. Given the significantly higher incidence of IGD in males compared with females, most existing research has centered on the male demographic (35). In other addictive disorders, sex differences have been widely discussed. For instance, a study using the stroop task found that male patients with cocaine use disorder may exhibit lower levels of FC in the cerebellum and brainstem compared to female patients (36). However, research on sex differences in IGD, particularly in the area of neuroimaging, remains relatively scarce. It is essential to delve deeper into the pathological characteristics and differences of IGD patients across different sexes. Male gender appears to be a risk factor for IGD (37, 38). Male online gamers have been shown not only to be more susceptible to developing IGD, but also to experience more severe impairments in cognitive function compared to females (39). On one hand, male gamers often find it more difficult to control their craving for gaming compared to female gamers, struggling to stop their gaming behavior (40). On the other hand, male gamers often exhibit a higher propensity for risk-taking, which makes them more attracted to game content related to violence, adventure, and gambling compared to females (41). The high craving and risk-taking tendencies in males lead to prolonged gaming sessions, making men more susceptible to IGD and also exposing them to a higher risk of physical health damage (42, 43). Although male gamers are more prone to IGD, female gamers can also experience adverse consequences due to excessive gaming behavior, exhibiting characteristics distinct from those of male IGD patients. Research has found that among problematic gamers with symptoms of ADHD, female players exhibit significantly higher levels of inattention compared to male players (44). In a study on smartphone addiction among adolescents in South Korea, researchers also found that attention deficits and self-control may play significant roles in regulating smartphone addiction and depression among female adolescents (45). Clearly, the impact of IGD on the social functioning of female gamers should not be underestimated.

The objective of this study is to explore whether there are differences in gaming behavior characteristics and sleep quality levels between patients with IGD and healthy individuals, as well as among patients of different sexes. Further fMRI research analyzed brain regions with differences in the ReHo index between male and female IGD patients compared to healthy subjects. Based on brain areas where interactions between diagnosis and sex were identified in the ReHo analysis, these regions served as seed points to further explore differences in FC across the whole brain between male and female IGD patients, aiming to identify potential neuroimaging biomarkers that distinguish between sexes in IGD. We also conducted partial correlation analyses between the FC values obtained from the aforementioned studies and patients’ gaming behavior characteristics, as well as their scores on sleep quality scales, to explore the relationships between sex-specific resting-state brain function activities, gaming behavior characteristics, and sleep quality. We hypothesize that changes in local brain activity and neural connectivity can be observed among IGD patients of different sexes. Furthermore, these alterations in neural connectivity may be associated with the duration of gaming, weekly gaming time, addiction scores, and sleep quality of the patients.

## 2 Materials and methods

### 2.1 Participants

One-hundred and two individuals aged between 18 and 40 years were included: 52 in the IGD group (31 males, 21 females) and 50 in the healthy control (HC) group (25 males, 25 females). Sex was defined as self-reported sex assigned at birth. Subjects were recruited between September 2022 and October 2023 via online advertisements in various communities and schools across Wuhan. IGD was diagnosed using the IGD-20 scale and DSM-5 diagnostic criteria. Potential participants first completed the IGD-20 scale online, and those scoring ≥71 were invited for further evaluation by experienced psychiatrists according to DSM-5 standards (46), where individuals meeting at least five of the nine DSM-5 criteria were classified as having IGD, a previously validated diagnostic threshold (47, 48). Renmin Hospital of Wuhan University’s Medical Ethics Committee approved the study protocol, which adhered strictly to the Declaration of Helsinki. All participants were fully briefed on the study’s aims and procedures and provided informed consent before participation. All participants received a stipend upon completion of all study procedures.

### 2.2 Clinical scale assessment

Essential demographic details were obtained from participants, including their sex, age, educational level, residence and average monthly income per capita. Gaming habits, including cumulative years spent gaming, weekly gaming duration and the ratio of monthly gaming expenditure to monthly disposable income, were documented. Data were also collected using validated clinical scales: (i) the Pittsburgh sleep quality inventory (PSQI) for self-evaluating sleep quality over the preceding month (49) using 19 items across seven dimensions (subjective sleep quality, sleep latency, sleep duration, habitual sleep efficiency, sleep disturbances, use of sleep medication, and daytime dysfunction), where the total score ranges from 0 to 21; (ii) the Hamilton depression rating scale (HAMD)-24 to assess depressive symptoms; (iii) the Hamilton anxiety rating scale (HAMA)-14 to assess anxiety; (iv) AUDIT) to determine levels of alcohol dependence; and (v) the Fagerström test for nicotine dependence (FTND) to evaluate nicotine dependence. Each participant underwent the mini-international neuropsychiatric interview (MINI) to rule out specific psychiatric conditions and confirm the accuracy of diagnoses.

### 2.3 Inclusion and exclusion criteria

#### 2.3.1 Inclusion criteria for the IGD group

① Aged 18-40, gender unrestricted; ② Meets greater than or equal to 5 DSM-5 criteria for IGD; ③ IGD-20 score ≥71; ④ At least middle school education.

#### 2.3.2 Inclusion criteria for the HC group

① Aged 18-40, gender unrestricted; ② Meets less than 5 DSM-5 criteria for IGD; ③ IGD-20 score <71; ④ At least middle school education.

#### 2.3.3 Exclusion criteria

① AUDIT score ≥8; ② FTND score ≥4;③ HAMD-24 score ≥8; ④ HAMA-14 score ≥7; ⑤ History of psychotropic medication and addictive medication use; ⑥ History of neurological or psychiatric disorders, chronic physical illness; ⑦ History of head trauma or presence of metal implants; ⑧ Left-handedness.

### 2.4 MRI acquisition

rs-fMRI data were collected using a 3T GE Signa HD×MRI scanner (General Electric, Brookfield, WI, USA). Prior to scanning, all participants were instructed to lie supine, close their eyes, and maintain a state of wakefulness and tranquility to reduce the impacts of head movements and mental activities. The scanning parameters for T1-weighted structural images were: repetition time (TR) 8.5 ms, echo time (TE) 3.2 ms, flip angle (FA) 12°, field of view (FOV) 256 × 256 mm, matrix size 256 × 256 mm, and voxel size at 1.0 × 1.0 × 1.0 mm. The slice thickness was 1.0 mm with no gaps, totaling 176 slices. For rs-BOLD fMRI, the echo planar imaging (EPI) sequence was TR 2000 ms, TE 30 ms, FA 90°, FOV 220 × 220 mm, matrix size 64 × 64 mm, voxel size 3.4 × 3.4 × 4.0 mm, slice thickness 4.0 mm, and gap 0 mm, producing a total of 36 slices and 240 volumes.

### 2.5 fMRI data processing

fMRI data were preprocessed on the MATLAB R2020b platform (MathWorks, Sherborn, MA, USA) utilizing the DPARSFA (Data Processing Assistant for Resting-State fMRI Analysis) toolbox. The procedure was as follows: (1) data format conversion: DICOM data were transformed into the NIFTI format for enhanced compatibility and ease of analysis; (2) initial signal stabilization: to account for the inherent instability of initial rs-fMRI signals, the first 10 volumes were excluded from the analysis; (3) slice timing correction: this step ensured temporal synchronization of signals across all brain regions by correcting the timing sequence of slices; (4) head motion correction: participants’ head movements were meticulously corrected, and subjects with maximum head displacement >3.0 mm or rotation >3.0° were excluded. Four participants were excluded due to their MRI data not meeting the required standards during the process of determining enrolled participants; (5) spatial normalization: data were spatially normalized to the Montreal Neurology Institute (MNI) space using an EPI template and resampled to 3 × 3 × 3 mm³; (6) signal regression analysis: regression analyses on 24 head motion parameters, global signals, white matter, and cerebrospinal fluid signals were conducted to further eliminate possible confounding factors; (7) detrending and band-pass filtering: linear trends were removed, and temporal band-pass filtering (0.01-0.08Hz) was applied to mitigate the influences of low-frequency drifts and high-frequency noise; (8) smoothing: post-smoothing processing was applied to the ReHo data.

### 2.6 Statistical analyses

SPSS v27.0 software (IBM Statistics, Armonk, NY, USA) was used to compare demographic, gaming characteristic information and clinical instrument indicators between groups. Bonferroni correction was used, with p < 0.05 as the statistical threshold for significant differences. SPM 12.0 toolbox was used for two-way ANOVA on ReHo signal values across four cohorts [considering diagnosis (IGD and HC) and sex (male and female)]. The analysis incorporated age, education level, and average frame displacement (FD) as covariates. Brain regions showing an interaction effect between sex and diagnosis from the ReHo analysis were selected as seed areas. Seed point masks were extracted using the Xjview toolbox on the MATLAB platform. Correlation coefficients between the time series of these seed areas and every other brain voxel were calculated to create correlation maps for each participant to illustrate brain connectivity. Fisher’s z transformation was applied to the correlation coefficients to enhance the data’s normal distribution. Two-way ANOVA was used to examine differences in FC between the identified seed points and the entire brain, considering diagnosis and sex as factors and controlling for age, education, and FD. Correction was performed using the Gaussian Random Field (GRF) method, with a voxel-level threshold of p < 0.001 and a cluster-level threshold of p < 0.05 for statistical significance.Partial correlation analysis was conducted with age and years of education as covariates. p < 0.05 as the statistical threshold for significant differences.

## 3 Results

### 3.1 Demographic, gaming and clinical characteristics

There were no statistically significant differences between male and female subjects in the IGD group and HC group in terms of age, gender, educational level, years of education, place of residence, and average monthly household income per capita (**Table 1**). Furthermore, there were no statistically significant differences in age and years of education between the IGD and the HC groups of different sexes. Detailed data are provided in the **Supplementary Materials**.

**Table 1:** Statistical results of the general demographic information of participants.

		IGD *n* (%)	HC *n* (%)	χ^2^/t	*P*
Gender	Male	31 (59.6%)	25 (50.0%)	0.952*^a^*	0.329
	Female	21 (40.4%)	25 (50.0%)		
Age (years)		23.35 ± 2.90	23.68 ± 2.40	0.632*^b^*	0.529
Level of education	High school or below	3 (5.8%)	2 (4.0%)	3.534^α^	0.171
	Bachelor	38 (73.1%)	29 (58.0%)		
	Master or higher	11 (21.2%)	19 (38.0%)		
Education (years)		16.63 ± 2.38	16.78 ± 2.05	0.329*^b^*	0.743
Residence	City	34 (65.4%)	40 (80.0%)	2.734*^a^*	0.098
	Rural or town	18 (34.6%)	10 (20.0%)		
Average monthly income per capita (yuan)	300∼1000	0 (0.0%)	2 (4.0%)		
	1000∼2000	6 (11.5%)	2 (4.0%)		
	2000∼5000	17 (32.7%)	23 (46.0%)	6.424*^a^*	0.170
	5000∼10000	19 (36.5%)	15 (30.0%)		
	>10000	10 (19.2%)	8 (16.0%)		

Normally distributed data are described as mean ± SD, while categorical data are presented as frequency (percentage). a: Statistical comparisons were conducted using the chi-square test; b: Statistical comparisons were conducted using the two-sample t-test. IGD, Internet gaming disorder; HC, Healthy control.

As expected, the weekly gaming time in the IGD group was significantly higher than in HC group [*P* < 0.001; F (3,98) = 44.114]. The number of gaming years differed between sexes, with male IGD participants reporting more years of gaming than female IGD participants [*P* = 0.029; F (3,98) = 4.880] (**Table 2**).

**Table 2:** Statistical results of participants’ gaming behavior information.

	IGD		HC		Main effect of diagnosis		Main effect of sex		Sex-diagnosis interaction	
	Males	Females	Males	Females	F	*P*	F	*P*	F	*P*
Number	31	21	25	25						
DSM-5	6.39±0.62	5.67 ± 0.80	1.12±0.83	1.84±1.77	436.910	<0.001	0.000	0.999	1.096	0.001
IGD-20	77.97±7.01	76.48 ± 5.61	47.00±16.16	46.08±19.88	129.236	<0.001	0.200	0.656	0.011	0.916
Gaming history (years)	13.97±3.98	12.24 ± 4.52	13.44±4.66	11.08 ±5.39	0.829	0.365	4.880	0.029	0.116	0.734
Gaming per week (hours)	25.98±10.04	28.19 ± 8.78	14.50±13.91	10.42±10.58	44.114	<0.001	0.181	0.672	2.037	0.157
Monthly gaming expenditure/Monthly disposable income	0.08±0.08	0.09 ± 0.13	0.09±0.18	0.04±0.07	1.071	0.303	0.726	0.396	1.134	0.290

The gaming characteristic information for the four groups is presented as mean ± SD. IGD, Internet gaming disorder; HC, Healthy control; DSM-5, Diagnostic and Statistical Manual of Mental Disorders-5.

IGD subjects scored significantly higher than HCs for HAMD-24 score [*P* = 0.012; F (3,98) = 6.621], HAMA-14 score [*P* = 0.007; F (3,98) = 7.606], PSQI total score [*P* = 0.007; F (3,98) = 7.720], sleep quality score[*P* = 0.005; F(3,98) = 8.369], sleep disturbances score[*P* = 0.014; F (3,98) = 6.290], and daytime dysfunction score[*P* = 0.013; F (3,98) = 6.353]. Habitual sleep efficiency score were also significantly different between male and female subjects [*P* = 0.006; F (3,98) = 7.757] (**Table 3**).

**Table 3:** Statistical results of participants’ clinical scale assessments.

	IGD		HC		Main effect of diagnosis		Main effect of sex		Sex-diagnosis interaction	
	Males	Females	Males	Females	F	*P*	F	*P*	F	*P*
Number	31	21	25	25						
FD	0.05 ± 0.02	0.06 ± 0.04	0.06 ± 0.02	0.06 ± 0.03	0.297	0.587	0.822	0.376	0.380	0.539
HAMD-24	6.48 ± 4.23	5.67 ± 3.76	4.00 ± 3.04	4.36 ± 3.46	6.621	0.012	0.096	0.757	0.639	0.426
HAMA-14	4.03 ± 2.92	3.62 ± 3.29	1.92 ± 2.36	2.72 ± 2.28	7.606	0.007	0.125	0.724	1.235	0.269
AUDIT	0.84 ± 1.74	0.86 ± 2.43	1.36 ± 1.96	0.16 ± 0.47	0.060	0.808	2.774	0.099	2.981	0.087
FTND	0.16 ± 0.90	0	0	0	0.659	0.419	0.659	0.419	0.659	0.419
PSQI-Total	7.16 ± 1.77	7.57 ± 2.40	5.96 ± 2.13	6.28 ± 2.70	7.720	0.007	0.662	0.418	0.010	0.920
Sleep quality	1.48 ± 0.51	1.29 ± 0.56	1.04 ± 0.61	1.00 ± 0.82	8.369	0.005	0.892	0.347	0.393	0.532
Sleep latency	1.35 ± 0.80	1.24 ± 0.83	1.16 ± 0.75	1.32 ± 0.90	0.119	0.731	0.017	0.895	0.714	0.400
Sleep duration	0.77 ± 0.56	0.90 ± 0.83	0.68 ± 0.56	0.76 ± 0.60	0.892	0.347	0.693	0.407	0.040	0.842
Habitual sleep efficiency	0.16 ± 0.37	0.57 ± 0.81	0.12 ± 0.33	0.32 ± 0.63	1.785	0.185	7.757	0.006	0.920	0.340
Sleep disturbances	1.16 ± 0.37	1.24 ± 0.44	1.04 ± 0.54	0.92 ± 0.40	6.29	0.014	0.061	0.806	1.263	0.264
Use of hypnotic medication	0.16 ± 0.52	0.00 ± 0.00	0.20 ± 0.71	0.04 ± 0.20	0.180	0.673	2.991	0.087	0.000	0.994
Daytime dysfunction	2.06 ± 0.68	2.33 ± 0.73	1.72 ± 0.68	1.92 ± 0.91	6.353	0.013	2.431	0.122	0.052	0.819

All values are mean ± SD. FD, frame displacement; IGD, Internet gaming disorder; HC, Healthy control; DSM-5, Diagnostic and Statistical Manual of Mental Disorders-5; HAMD-24, 24-item Hamilton depression rating scale; HAMA-14, 14-item Hamilton anxiety rating scale; AUDIT, the alcohol use disorders identification test; FTND, the Fagerström test for nicotine dependence; PSQI, Pittsburgh sleep quality index.

### 3.2 ReHo: differential brain activity in IGD and HC subjects according to sex

**Figure 1:**
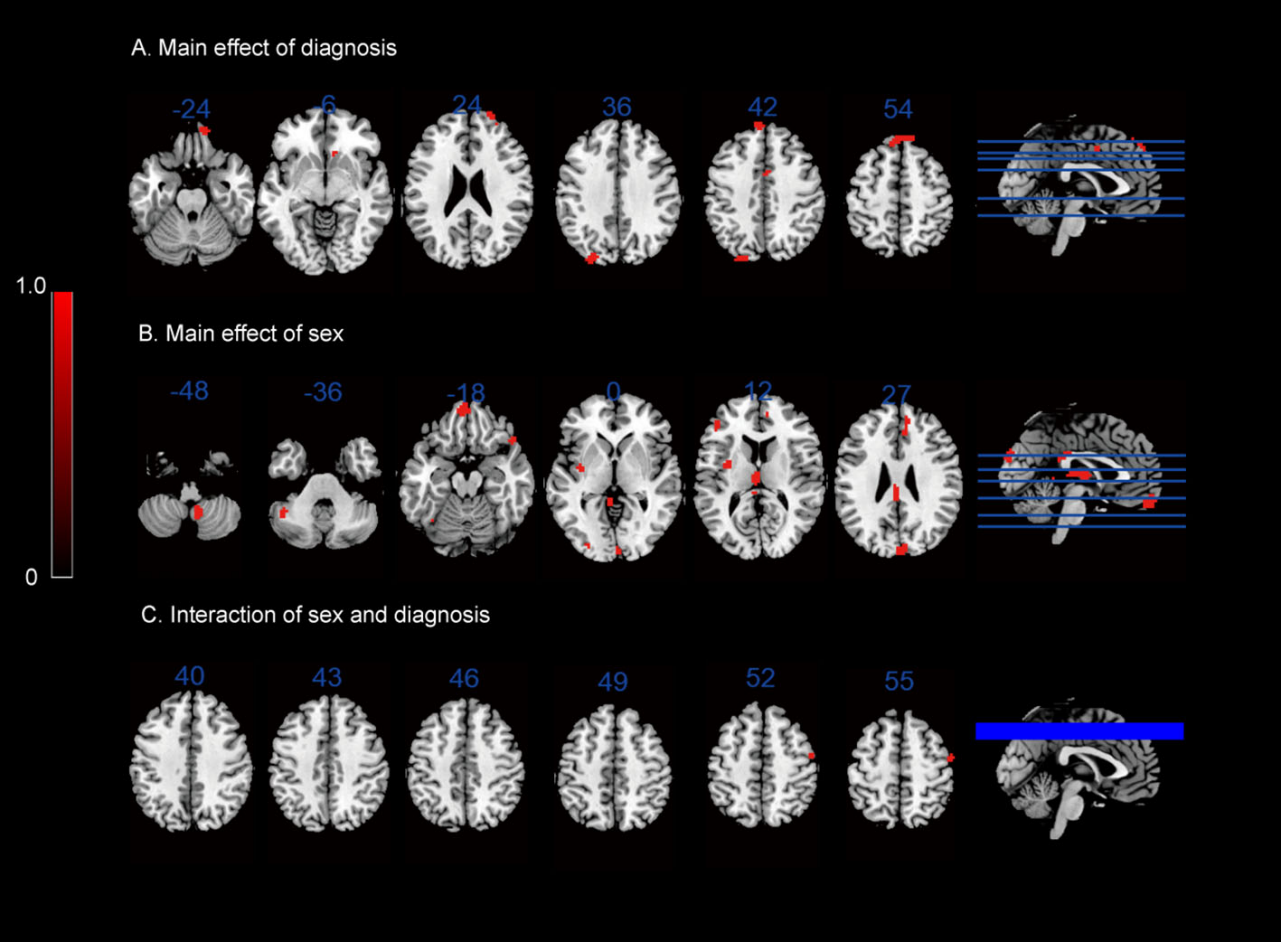
ReHo results: **(A)** main effect of diagnosis on the ReHo maps; **(B)** main effect of sex on the ReHo maps; **(C)** interaction between sex and diagnosis on the ReHo maps.

In terms of the main effect of diagnosis (**Table 4**, **Figure 1A**), compared with HCs, IGD subjects exhibited changes in ReHo in the right medial OFC, right caudate nucleus, rSFG, rMFG, left superior occipital gyrus, lSFG, and rSMA. In terms of the main effect of sex (**Table 4**, **Figure 1B**), male and female subjects showed significant differences in ReHo in the left gyrus rectus, left medial dorsal nucleus of the thalamus, left inferior frontal gyrus (triangular part), right anterior cingulate cortex (ACC), and cuneus. Regarding the interaction between diagnosis and sex (**Table 4**, **Figure 1C**), there were significant differences in ReHo in the rMFG across the four groups.

**Table 4:** ReHo differences between IGD and HC subjects for different sexes.

Comparisons	Cluster	Region	BA	Voxels	MNI Coordinates			Peak intensity
					x	y	z	
Main effect of diagnosis								
	Cluster1	R Medial Orbitofrontal Cortex	11	22	12	51	-21	20.526
	Cluster2	R Caudate Nucleus	NA	18	9	24	-6	14.959
	Cluster3	R Superior Frontal Gyrus	10	21	24	63	24	13.912
		R Middle Frontal Gyrus						
	Cluster4	L Superior Occipital Gyrus	19	40	-24	-84	36	18.690
	Cluster5	L Superior Frontal Medial Gyrus	8	94	-6	54	45	17.093
	Cluster6	R Supplementary Motor Area	32	19	0	3	45	16.890
		L Supplementary Motor Area						
Main effect of sex								
	Cluster1	R Cerebellum Inferior	NA	27	9	-54	-45	20.256
	Cluster2	L Cerebellum Superior	NA	30	-45	-54	-33	16.606
	Cluster3	L Gyrus Rectus	11	96	-3	60	-24	30.456
	Cluster4	R Superior Temporal Pole	38	23	51	24	-15	24.342
	Cluster5	L Insula	13	33	-36	-6	-3	21.335
	Cluster6	L Inferior Occipital Gyrus	18	25	-33	-84	-6	18.416
	Cluster7	L Calcarine Fissure	18	26	6	-93	6	18.943
	Cluster8	L Medial Dorsal Nucleus of the Thalamus	29	113	-3	-12	6	27.999
	Cluster9	L Inferior Frontal Gyrus, Triangular Part	46	37	-45	36	15	18.889
	Cluster10	R Anterior Cingulate Cortex	9	40	9	45	24	18.320
	Cluster11	L Cuneus	19	48	3	-93	27	33.526
		R Cuneus						
	Cluster12	L Posterior Cingulate Cortex	23	30	-3	-30	27	19.759
Sex-diagnosis interaction								
	Cluster1	R Middle Frontal Gyrus	6	14	54	-6	54	14.946

R, Right; L, Left; MNI, Montreal Neurological Institute; BA, Brodmann’s area; NA, Not Applicable.

### 3.3 FC patterns in IGD: impact of diagnosis and sex differences

**Figure 2:**
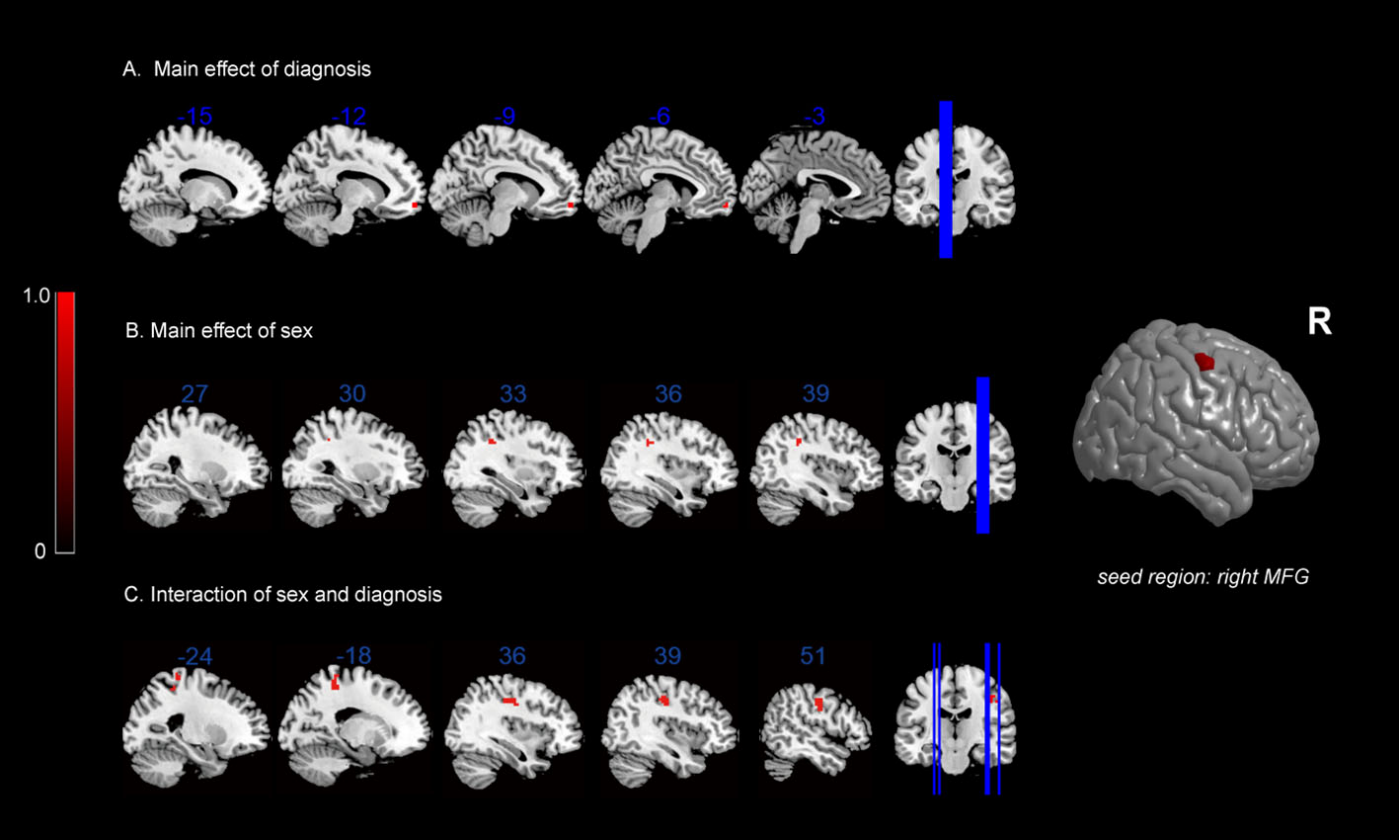
FC results: **(A)** main effect of diagnosis on FC maps; **(B)** main effect of sex on FC maps; **(C)** interaction between sex and diagnosis on FC maps.

**Figure 3:**
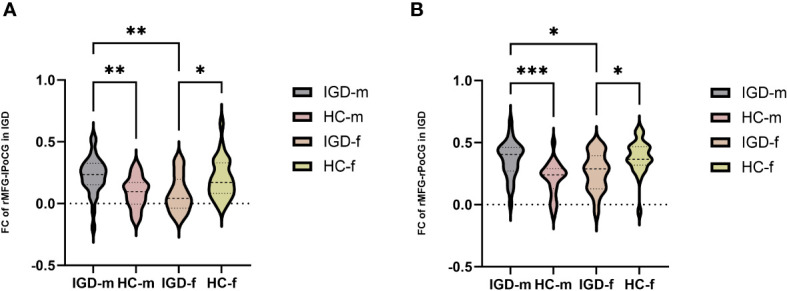
Post-hoc analysis of FC results based on the interaction between diagnosis and sex. **(A)** Comparison of FC values in the rMFG and left PoCG among the four groups. **(B)** Comparison of FC values in the rMFG and right PoCG among the four groups. *: p < 0.05, **: p < 0.01, ***: p < 0.001 (most significant).

In terms of the main effect of diagnosis (**Table 5**, **Figure 2A**), changes in FC were observed between the rMFG and the left medial OFC in IGD subjects compared with HCs. Regarding the main effect of sex (**Table 5**, **Figure 2B**), male and female IGD subjects showed differences in FC between the rMFG and both the right inferior parietal lobule and the right supramarginal gyrus. In terms of the interaction effect between diagnosis and sex (**Table 5**, **Figure 2C**), there were significant differences in FC between the rMFG and both the right and left PoCG between IGD males and females compared with HCs. In the post-hoc analysis (**Figure 3**), male IGD subjects had higher FC values between the rMFG and both the left and right PoCG compared with female IGD subjects. Additionally, male and female IGD subjects exhibited opposite FC patterns. The FC values of male IGD subjects were greater than those of HC males, while the FC values of female IGD subjects were less than those of HC females.

**Table 5:** Differences in the rMFG FC between the IGDs and the HCs.

Seed	Comparisons	Region	BA	Voxels	MNI Coordinates			Peak intensity
					x	y	z	
rMFG	Main effect of diagnosis	L Medial Orbitofrontal Cortex	11	11	-9	60	-12	17.010
	Main effect of sex	R Inferior Parietal Lobule	NA	19	36	-39	36	18.629
		R Supramarginal Gyrus						
	Sex-diagnosis interaction	R Postcentral Gyrus	4	69	39	-21	39	25.905
		L Postcentral Gyrus	3	34	-21	-39	69	19.416

R, right; L, left; MNI, Montreal Neurological Institute; BA, Brodmann’s area; rMFG, right middle frontal gyrus; NA, Not Applicable.

### 3.4 Partial correlation analysis

In the male IGD group, the FC values between the rMFG and the left PoCG were significantly correlated with the daytime dysfunction score [r = 0.555, *P* = 0.002] and the PSQI total score [r = 0.536, *P* = 0.003]. Additionally, the FC values between the rMFG and the right PoCG showed significant positive correlations with the daytime dysfunction score [r = 0.582, *P* = 0.001] and the PSQI total score [r = 0.466, *P* = 0.011]. In the female IGD group, the FC values between the rMFG and the right PoCG were significantly correlated with gaming hours per week [r = 0.505, P = 0.027] and the sleep disturbances score [r = 0.582, *P* = 0.009], while the FC values between the rMFG and the left PoCG showed no significant correlations with scores on clinical scales.

**Figure 4:**
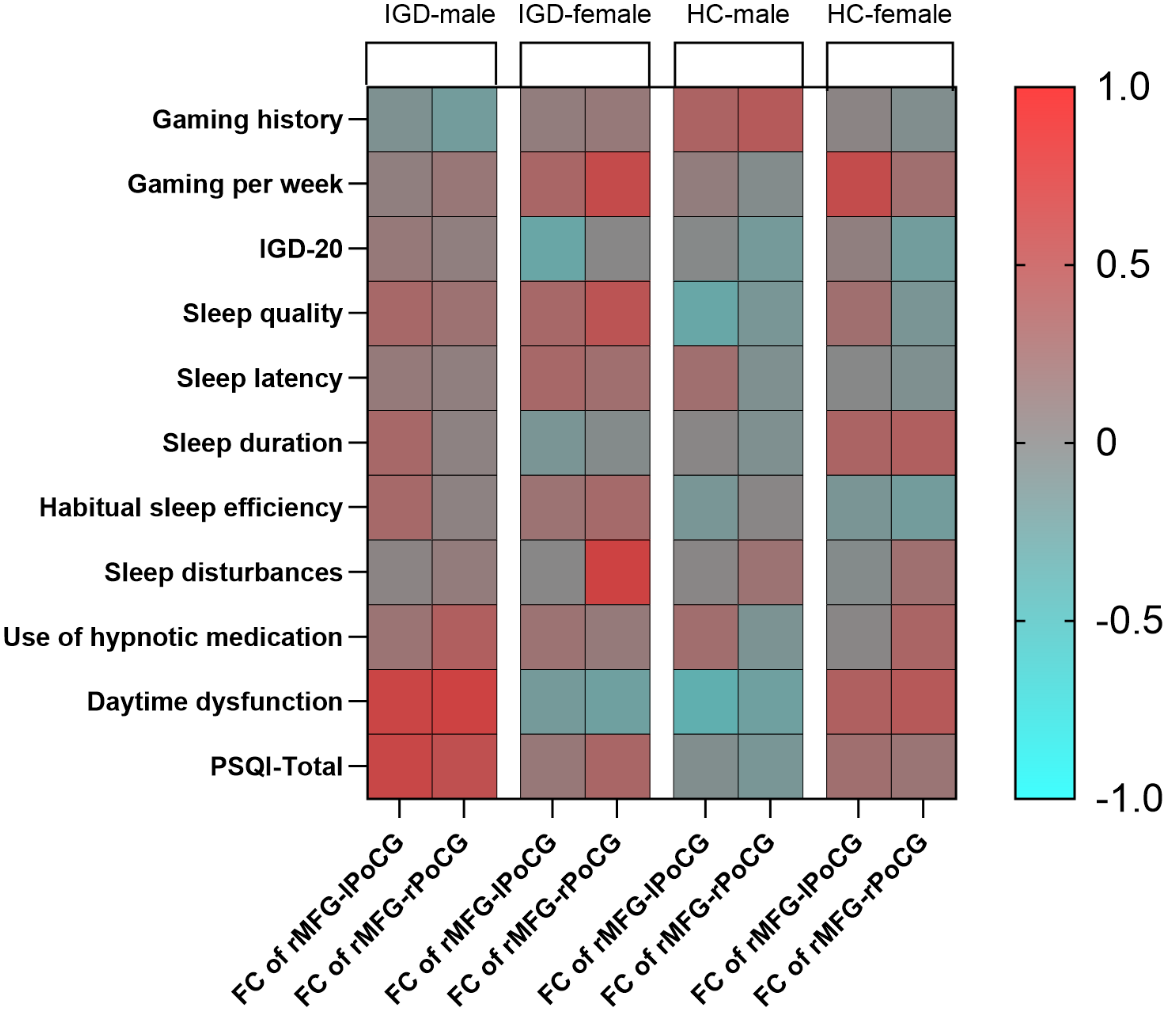
Partial correlation analysis heatmap (r-values).

In the healthy male group, the FC values between the rMFG and bilateral PoCG showed no significant correlations with scores on clinical scales. Similarly, no significant correlation was found between the FC values of the rMFG and right PoCG and any clinical scales in the healthy female group. However, the FC values between the rMFG and left PoCG was positively correlated with weekly gaming time in the healthy female group [r = 0.491, *P* = 0.017] (**Figure 4****).** **Supplementary Materials** include detailed tables of the partial correlation analyses for the four groups.

We utilized a t-test to assess the significance of the correlation coefficients derived from the sample to determine their generalizability to the overall population. The results indicated that the p-value for the significance test of the correlation between FC and the PSQI total score, involving the rMFG and the left PoCG in the male IGD group, was 0.002. In contrast, the p-value for the female IGD group for the same regions was 0.127. Additionally, the p-value for the correlation between FC and the PSQI total score involving the rMFG and the right PoCG in the male IGD group was 0.008, while the corresponding p-value in the female IGD group was 0.228. The current results suggest that the correlations between FC and PSQI involving the rMFG and bilateral PoCG are significant in the male IGD group, whereas the results for the female group are not significant.

### 3.5 Power analysis

**Figure 5:**
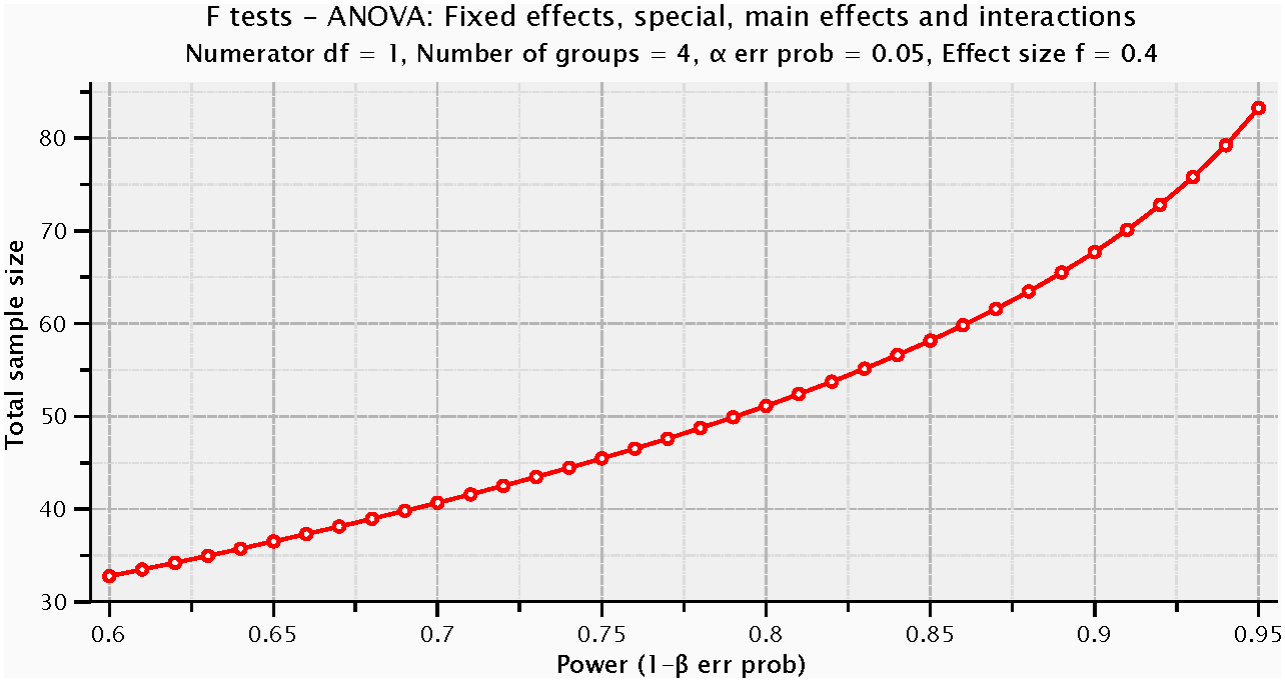
Power analysis.

For *a priori* power analysis, we employed G*Power (version 3.1.9.7) and selected the “ANOVA: Fixed effects, special, main effects and interactions” to assess interactions based on conventions in existing literature (50). We set the effect size (f) at 0.40, alpha error probability at 0.05, and power (1-β error probability) at 0.80, with numerator degrees of freedom set to 1 and the number of groups to 4. The calculation indicated that approximately 52 participants were needed in total, with about 13 participants per group on average. The actual sample size exceeded the minimum suggested by the power analysis, enhancing the statistical stability and reliability of our study results (**Figure 5**).

## 4 Discussion

This study revealed notable sex-specific differences in the neurological basis of IGD according to ReHo and FC measures. Crucially, compared to HCs, male and female IGD participants demonstrated significant alterations in local brain activity within the rMFG. Significant differences were found in the FC between the rMFG and bilateral PoCG among male and female IGD patients compared to their respective healthy control groups. Specifically, male IGD patients exhibited higher FC values compared to healthy male controls, while female IGD patients showed lower FC values than healthy female controls. Additionally, the FC values between the rMFG and bilateral PoCG were higher in male IGD patients compared to female IGD patients. These findings suggest that the ReHo of the rMFG and changes in FC between the rMFG and bilateral PoCG could serve as potential neuroimaging biomarkers to distinguish brain functional activities in IGD patients of different sexes.

The neural mechanisms underlying why males are more prone to developing IGD than females remain unclear (39). Previous research has demonstrated that neural alterations associated with risky decision-making may exist exclusively in male IGD individuals (51). Moreover, activity patterns in the frontoparietal network (FPN) of IGD patients appear to be differ from those in healthy individuals. Male IGD patients exhibit more coherent and unified structural or functional alterations in the FPN, a pattern not observed in their female counterparts (52, 53). Positioned in the lateral segment of the frontal lobe, the rMFG is a key component of the DMN (54). The DMN is increasingly recognized as a vital network for predicting IGD outcomes (55). Current theories suggest that abnormal interactions among the executive control network, the DMN, and the salience network are key features of addiction (56). Impairments in the frontal cortex, where the rMFG is located, have been proposed as a potential mechanism underlying IGD (57). The rMFG plays an essential role in executive control functions, which encompass working memory, attention, problem-solving, and decision-making (58). Our study results suggest that abnormal local brain activity in the rMFG may be a key region contributing to the greater susceptibility of males to develop IGD compared to females.

The MFG also plays a crucial role in the development and progression of other addictive disorders. The neurobiological mechanisms of addictive disorders often show considerable overlap (59). For instance, neural activity and cognitive functions in IGD share similarities with those in gambling disorder (60), with pathological gambling associated with increased connectivity between the rMFG and the right striatum and decreased connectivity to other frontal regions (61). Similarly, active substance addicts, particularly who use nicotine, also exhibit reduced activity in the rMFG (62). The conclusions of this study corroborate the presence of certain overlaps in the neural mechanisms between IGD and other addictive disorders.

In this study, an increase in FC between the rMFG and bilateral PoCG was observed in male IGD patients compared to female IGD patients. This indicates that enhanced neural activity between the rMFG and bilateral PoCG plays a significant role in influencing the onset and progression of IGD in different sexes.The PoCG, situated on the parietal lobe’s lateral surface and known as the primary somatosensory cortex, plays a crucial role in processing various bodily sensations like touch, pressure, temperature, and pain (63). ReHo metrics for the right and left PoCG have been shown to predict IGD severity (64). Both the MFG and the PoCG are part of the sensorimotor network. The sensorimotor network is primarily responsible for processing and interpreting sensory information (such as touch, pain, and temperature) received from various parts of the body. It coordinates sensory input with motor output, ensuring that motor behaviors are adapted to sensory signals (65). Previous studies have shown that IGD is marked by an enhanced sensorimotor network (66). A previous exploratory study found that male online gamers, compared to females, prefer first-person or third-person shooter games and competitive, achievement-oriented, and aggressive online games. The process of playing these games typically requires coordination between brain regions responsible for decision-making control and bodily perception (41). Combining the results of this study suggests that the connection between brain regions responsible for decision-making control and bodily perception functions is tighter in male IGD patients compared to female gamers. This may confer a performance advantage to male players in action shooter games over females.

In other addiction studies, researchers using independent component analysis have found that connectivity within the sensorimotor network is significantly negatively correlated with scores for social media addiction and smartphone addiction (67). Another study found that smokers exhibited enhanced FC between the posterior part of the nucleus accumbens and regions associated with the sensorimotor network (68). Previous research has identified enhanced FC between the subcortical networks and motor networks in individuals with IGD and tobacco use disorder, highlighting the close relationship between the reward system and behavior in addicted individuals (69). Our study also suggests a significant association between the inhibitory control system and behavior.

Our study also detected opposite FC patterns between male and female IGD subjects, with increased FC in males with IGD compared with the control group and decreased FC in females with IGD. The findings of the study indicate that male and female brains may exhibit different neurobiological responses to IGD. Previous research has also observed that changes in the resting-state brain function of IGD patients of different sexes show opposite trends. For example, Wang et al. (70) reported similar findings, where male IGD subjects showed increased ReHo values in the left middle occipital gyrus (lMOG) and right middle temporal gyrus (rMTG) compared with the control group.Conversely, female IGD subjects had reduced ReHo values in the lMOG and rMTG. Another study indicated the potential value of measuring addiction-related brain networks (involving the frontal lobe, ACC, and striatum) to distinguish IGD from healthy individuals, with opposing trends in FC strength patterns between sexes (71). Specifically, male IGD patients exhibit increased FC both within and between addiction-related brain networks, whereas female IGD patients show decreased FC within and between these networks. Dong et al. (72), in a task-state fMRI study, found distinct FC patterns (related to the DLPFC and striatum) in brain regions related to executive control during gaming and forced interruption in male gamers and greater differences in these FC patterns between female IGD subjects and HCs during the forced interruption period. These findings suggest significant sex-specific effects in advanced cognitive functions such as executive control in IGD.

Our study found a significant positive correlation between the FC of the rMFG and bilateral PoCG and the PSQI total score in male IGD subjects. However, no such correlation was found in the female IGD group, or in the healthy male and female groups. Although the difference in this correlation is not significant between male and female IGD patients, it indicates that the FC of the rMFG and bilateral PoCG is more closely associated with sleep quality in male IGD patients. Previous research on sleep quality changes in IGD patients has been relatively scarce. Zheng et al. (73) found a positive correlation between the FC of the right posterior hippocampus and left caudate nucleus and the PSQI total score in IGD patients, which serves as an indirect mediator between sleep quality and IGD addiction severity. Niu et al. (15) found a negative correlation between the dReHo value of the left caudate nucleus and the PSQI total score. Our study, however, shows that the FC between the rMFG and bilateral PoCG plays an important role in the sleep quality of male IGD patients, expanding on previous findings.

Currently, existing research has demonstrated a close association between the PoCG and an individual’s level of sleep quality. A study of healthy young males detected a positive correlation between PSQI total score and increased FC of the bilateral postcentral gyrus, indicating a possible association between poor sleep quality and increased connectivity of sensory and somatomotor functions during rest (74). The PoCG’s overactivity could be a significant factor related to insomnia-associated anxiety (75). Extended periods of internet gaming may drastically disrupt normal sleep patterns by intensifying this FC, meriting further detailed examination. Moreover, increased internet use has been shown to adversely affect physical activity and nutritional status, subsequently increasing the risk of sleep disorders (76). Sleep quality is not solely a consequence of IGD but could potentially exacerbate the symptoms and severity of IGD, though the exact mechanisms remain undefined. Emerging evidence suggests that sleep disruption events might influence levels of brain damage biomarkers in individuals with internet addiction (77). Some studies have proposed that sleep quality indirectly impacts the degree of internet addiction through depression levels (78).

In summary, this study has identified potential neuroimaging biomarkers that influence local brain activity and neural connectivity in IGD patients of different sexes. The findings reveal significant differences in neural activity within brain regions responsible for inhibitory control and somatosensory perception in male and female IGD patients. These differences will aid in the development of personalized medical interventions, enhancing the effectiveness of these measures. Additionally, the results of this study will facilitate the creation of educational programs tailored to the characteristics of male and female IGD patients.

This study has some limitations. First, our sample size was relatively small, and participants were primarily students from local universities in Wuhan, which may not fully represent the wider population. The limited number of female IGD participants in our sample may have affected the reliability of the partial correlation analysis results for the female IGD group. In future work, we intend to reanalyze and verify our findings by increasing the sample size. Second, this study was cross-sectional, which does not robustly establish causal relationships between the different variables studied. Future research is needed to explore the long-term impacts of IGD on mental health, cognitive function, and social behavior. Lastly, our study explores sex differences in the neurobiological mechanisms underpinning IGD, but it does not fully reveal the complex network interactions and working modes during the formation of IGD. Future studies should conduct multimodal imaging research combining fMRI with other imaging techniques such as PET, EEG, or structural MRI to provide more comprehensive information on the brain function and structure of IGD patients.

## 5 Conclusion

Our study provides new insights into sex-specific neural mechanisms underlying IGD. Our findings demonstrate that the rMFG plays a critical role in the manifestation and maintenance of IGD, with its neural connectivity patterns differing between sexes. In male IGD subjects, there was increased FC of the rMFG with bilateral PoCG, and this enhanced FC was positively correlated with PSQI total score. Female IGD subjects, on the other hand, exhibited an opposite pattern of FC. These findings emphasize the importance of considering sex differences in the neural basis of IGD. The sex-specific changes in FC patterns, especially those involving the rMFG, offer valuable insights for potential targeted therapeutic interventions.

## Data availability statement

The raw data supporting the conclusions of this article will be made available by the authors, without undue reservation.

## Ethics statement

The studies involving humans were approved by the Clinical Research Ethics Committee of Wuhan University Renmin Hospital (Ethical Review No.WDRY2022-K090). The studies were conducted in accordance with the local legislation and institutional requirements. The participants provided their written informed consent to participate in this study.

## Author contributions

MZ: Data curation, Investigation, Visualization, Writing – original draft. GG: Data curation, Methodology, Project administration, Supervision, Writing – review & editing. BR: Formal Analysis, Methodology, Writing – review & editing. HZ: Software, Writing – review & editing. JH: Data curation, Writing – review & editing. NT: Resources, Writing – review & editing. LB: Resources, Writing – review & editing. LX: Conceptualization, Project administration, Supervision, Writing – review & editing. GW: Conceptualization, Funding acquisition, Supervision, Writing – review & editing.
