# Supplementary material for: Sex differences of neural connectivity in internet gaming disorder and its association with sleep quality: an exploratory fMRI study
Source: Front Psychiatry. 2024 May 30;15:1379259. doi: 10.3389/fpsyt.2024.1379259 (PMC11169786; doi:10.3389/fpsyt.2024.1379259)
Supplement: Supplementary file 1 [file Table_1.docx]

Supplementary Material

**Sex differences of neural connectivity in internet gaming disorder and its association with sleep quality: an exploratory fMRI study**

Mingzhe Zhou, Guoqing Gao, Bei Rong, Haomian Zhao, Junhua Huang, Ning Tu, Lihong Bu, Ling Xiao*, Gaohua Wang*

*** Correspondence:**Ling Xiao lingxiaoxiao@whu,edu.cn

Gaohua Wang [wgh6402@whu.edu.cn](mailto:wgh6402@whu.edu.cn)

# Supplementary Figures and Tables

## Supplementary Tables

| **Table S1** Comparison of demographic results between IGD patient groups and HC groups according to sex | | | | | | | | | | |
| --- | --- | --- | --- | --- | --- | --- | --- | --- | --- | --- |
|  | IGD | | HC | | Main effect of diagnosis | | Main effect of sex | | Sex-diagnosis interaction | |
|  | Males | Females | Males | Females | F | *P* | F | *P* | F | *P* |
| Number | 31 | 21 | 25 | 25 |  |  |  |  |  |  |
| Age (years) | 23.48±3.08 | 23.14±2.65 | 23.36±2.36 | 24.00±2.45 | 0.468 | 0.495 | 0.078 | 0.781 | 0.838 | 0.362 |
| Education (years) | 16.32±2.36 | 17.10±2.41 | 16.72±1.79 | 16.84±2.32 | 0.025 | 0.874 | 0.999 | 0.320 | 0.534 | 0.467 |

**Note:** Normally distributed data are described as mean ± SD. **Abbreviations:** IGD: Internet gaming disorder; HC: Healthy control.

**Table S2** Results of partial correlation analysis

|  | | IGD-male | | IGD-female | | HC-male | | HC-female | |
| --- | --- | --- | --- | --- | --- | --- | --- | --- | --- |
|  | | FC of rMFG-lPoCG | FC of rMFG-rPoCG | FC of rMFG-lPoCG | FC of rMFG-rPoCG | FC of rMFG-lPoCG | FC of rMFG-rPoCG | FC of rMFG-lPoCG | FC of rMFG-rPoCG |
| Gaming history（years） | r | -0.085 | -0.172 | 0.087 | 0.118 | 0.305 | 0.379 | 0.020 | -0.060 |
|  | *P* | 0.660 | 0.371 | 0.724 | 0.630 | 0.157 | 0.075 | 0.929 | 0.785 |
| Gaming per week（hours） | r | 0.061 | 0.143 | 0.275 | 0.505 | 0.084 | -0.039 | 0.491 | 0.194 |
|  | *P* | 0.753 | 0.459 | 0.255 | **0.027** | 0.702 | 0.860 | **0.017** | 0.374 |
| IGD-20 | r | 0.120 | 0.063 | -0.262 | -0.001 | -0.023 | -0.154 | 0.065 | -0.191 |
|  | *P* | 0.534 | 0.745 | 0.279 | 0.997 | 0.919 | 0.484 | 0.768 | 0.382 |
| Sleep quality | r | 0.256 | 0.181 | 0.255 | 0.434 | -0.265 | -0.127 | 0.196 | -0.108 |
|  | *P* | 0.181 | 0.348 | 0.292 | 0.063 | 0.221 | 0.563 | 0.370 | 0.622 |
| Sleep latency | r | 0.101 | 0.065 | 0.269 | 0.207 | 0.195 | -0.067 | -0.010 | -0.074 |
|  | *P* | 0.602 | 0.737 | 0.266 | 0.395 | 0.373 | 0.760 | 0.963 | 0.736 |
| Sleep duration | r | 0.262 | 0.042 | -0.106 | -0.035 | 0.019 | -0.073 | 0.292 | 0.338 |
|  | *P* | 0.169 | 0.829 | 0.666 | 0.885 | 0.931 | 0.741 | 0.176 | 0.115 |
| Habitual sleep efficiency | r | 0.249 | 0.05 | 0.163 | 0.243 | -0.127 | 0.013 | -0.107 | -0.183 |
|  | *P* | 0.194 | 0.796 | 0.505 | 0.316 | 0.562 | 0.953 | 0.627 | 0.404 |
| Sleep disturbances | r | 0.023 | 0.095 | 0.003 | 0.582 | 0.009 | 0.175 | -0.032 | 0.188 |
|  | *P* | 0.906 | 0.623 | 0.989 | **0.009** | 0.969 | 0.424 | 0.883 | 0.391 |
| Use of hypnotic medication | r | 0.156 | 0.335 | 0.164 | 0.110 | 0.208 | -0.101 | 0.014 | 0.281 |
|  | *P* | 0.418 | 0.075 | 0.102 | 0.275 | 0.340 | 0.645 | 0.949 | 0.193 |
| Daytime dysfunction | r | 0.555 | 0.582 | -0.158 | -0.204 | -0.336 | -0.202 | 0.324 | 0.391 |
|  | *P* | **0.002** | **0.001** | 0.518 | 0.401 | 0.117 | 0.356 | 0.132 | 0.065 |
| PSQI-Total | r | 0.536 | 0.466 | 0.127 | 0.275 | -0.064 | -0.126 | 0.196 | 0.147 |
|  | *P* | **0.003** | **0.011** | 0.605 | 0.254 | 0.771 | 0.567 | 0.370 | 0.502 |

Note: r: partial correlation coefficient; P: significance level (two-tailed). Abbreviations: IGD: Internet gaming disorder；HC: Healthy control; FC: functional connectivity；MFG: middle frontal gyrus; PoCG: postcentral gyrus; PSQI: Pittsburgh sleep quality index；
